# Characterization of *4-Coumarate-CoA Ligase* (*4CL)* Genes in Wheat Uncovers *Ta4CL91*’s Role in Drought and Salt Stress Adaptation

**DOI:** 10.3390/plants14091301

**Published:** 2025-04-25

**Authors:** Ze Zhang, Xiuli Yang, Dongxian Ning, Rong Li

**Affiliations:** Institute of Wheat Research, Shanxi Agricultural University, Linfen 041000, China; zhangzelab3027@163.com (Z.Z.); yangxiuli1234@163.com (X.Y.)

**Keywords:** wheat, 4CL, VIGS, abiotic stress

## Abstract

During the growth process, wheat (*Triticum aestivum* L.) is frequently subjected to abiotic stress. However, the mechanisms of abiotic stress in wheat are not yet well understood. In other crops, 4-coumarate-CoA ligases (4CLs) have been found to be involved in abiotic stress responses, but a systematic analysis of the response of *4CLs* to abiotic stress in wheat has not yet been conducted. Through a comprehensive genome-wide analysis, we identified 110 putative *4CL* genes in wheat. These genes were phylogenetically divided into distinct groups, with the authentic *4CLs* forming a separate branch and *4CL-like* genes being further categorized into six subgroups. Each clade exhibited conserved gene structures and motif compositions. Promoter analysis identified a variety of stress-responsive cis-regulatory elements within *Ta4CL* genes, indicating their potential involvement in stress regulation mechanisms. Expression profiling under drought and salt stress conditions identified specific *4CL* genes linked to stress tolerance. Notably, *Ta4CL91*, a member of the 4CL clade, showed strong dual responsiveness to both drought and salt stresses. Using virus-induced gene silencing (VIGS), we suppressed *Ta4CL91* expression and observed that the *Ta4CL91*-silenced plants became more sensitive to drought and salt stresses, highlighting *Ta4CL91*’s critical role in stress adaptation in wheat. This comprehensive study not only expands our understanding of the *4CL* gene family in wheat but also highlights the critical involvement of specific *4CL* members, such as *Ta4CL91*, in mediating this plant’s resistance to abiotic stresses.

## 1. Introduction

Wheat is one of the world’s major staple crops, providing essential carbohydrates and proteins for humanity and serving as a primary food source in many countries [1]. However, wheat production is often affected by abiotic stresses such as drought, high temperatures, salinity, and low temperatures, which significantly reduce its growth and yield [2,3]. Climate change has intensified the frequency of extreme weather events, further threatening the stability and productivity of wheat [4]. Therefore, developing stress-resistant varieties and improving cultivation techniques are crucial for ensuring global food security.

4-coumarate-CoA ligases (4CL, EC 6.2.1.12) are the key enzymes in the phenylpropanoid pathway, serving as the main branch point enzymes. They catalyze the conversion of cinnamic acid into corresponding CoA thioesters [5]. The *4CL* genes have the capacity to encode multiple enzymes and demonstrate distinct substrate affinities, which appear to align with various metabolic functions [6]. The compounds produced by 4CL serve as critical precursors for a diverse array of enzymes that drive the biosynthesis of numerous biologically and structurally significant substances, including lignin, anthocyanins, flavonoids, aurones, suberin, cutin, coumarins, stilbenes, sporopollenin, and other essential metabolites [5].

The 4CL proteins are characterized by the presence of two highly conserved domains: Box I, with the amino acid sequence SSGTTGLPKGV, and Box II, with the amino acid sequence GEICIRG [7]. Box I represents the AMP-binding functional domain, a pivotal component that is universally preserved across all members of the adenylate-forming enzyme family [7]. The Box II region exhibits a high degree of conservation specifically within the 4CL enzyme and does not play a direct role in catalysis [8,9]. In accordance with *De Azevedo Souza*’s classification, 104 proteins associated with 4CL have been classified into two overarching groups [10]. The first group encompasses a substantial cohort of adenylate-forming enzymes that potentially have vital metabolic functions and are conserved across a wide range of organisms [10]. The second group comprises adenylate-forming proteins, which include both authentic 4CL proteins and previously annotated *Arabidopsis* 4CL-like acyl-CoA synthetase (ACS) proteins [10]. In *Arabidopsis thaliana*, At4CL1–4 has been identified as the authentic 4CL and is further classified into two distinct types [11,12]. Specifically, At4CL1, At4CL2, and At4CL4 are grouped under type I (associated with lignin biosynthesis), while At4CL3 is situated within the type II cluster (linked to phenylpropanoid biosynthesis other than lignin) [12].

Existing reports indicate that the functions of the *4CL* genes are intricately intertwined with the environmental pressures that plants confront [13,14]. A reduction in stress was primarily accomplished by regulating the biosynthesis of lignin, flavonoids, and other secondary metabolites [15,16,17,18]. *Ta4CLs* respond to UV radiation, hormonal signals, and environmental stimuli [19]. 6-BA promoted lignin accumulation in wheat cell walls and enhanced 4CL activity and *Ta4CL* gene expression under waterlogging stress [20]. In *Fraxinus mandshurica*, overexpression of *Fm4CL2* conferred enhanced drought tolerance in tobacco [21]. The overexpression of *Fm4CL-like1* in tobacco led to an enhancement in drought tolerance, attributed to the heightened accumulation of lignin and increased activities of antioxidant enzymes [22]. In the context of Mulberry, it was observed that all four *Ma4CLs* exhibited a response to salt stress. It was noted that *Ma4CL1–3* displayed an overall up-regulation in the presence of salt stress, whereas *Ma4CL4* demonstrated a pattern of up-regulation in stems and down-regulation in roots following exposure to salt stress [23]. In cotton plants, *Gh4CL7*-gene-silenced strains exhibited heightened sensitivity to drought treatment. Conversely, the overexpression of *Gh4CL7* in *Arabidopsis* lines resulted in heightened tolerance of drought stress [24]. Despite the plethora of prior research, our understanding of the molecular mechanisms underlying the effects of 4CL on various stresses remains limited. Exploring the gene expression patterns of *4CL* genes under different stress conditions could provide valuable insights for further elucidating the functional significance of *4CLs* in wheat.

In this study, we adopted a unified 4CL nomenclature to designate both *4CL* and *4CL-like* genes. Through an extensive genome-wide analysis, we identified 110 *Ta4CL* genes and conducted detailed analyses of their protein structures, gene architectures, and promoter region cis-acting elements. By examining the transcriptional changes of *Ta4CLs* under drought and salt stress conditions, we identified *Ta4CL91* as a gene significantly upregulated in response to these stressors. Functional validation through virus-induced gene silencing demonstrated that *Ta4CL91*-knockdown plants displayed reduced stress tolerance, highlighting this gene’s essential role in stress adaptation. These results establish *Ta4CL91* as a promising candidate for breeding stress-resistant wheat varieties and offer significant insights into the functional roles of *Ta4CLs*.

## 2. Results

### 2.1. Genome-Wide Identification and Phylogenetic Analysis of 4CL Genes in Wheat

Based on the genome-wide data of bread wheat from Ensembl plants, two hidden Markov models corresponding to the AMP-binding (PF00501) and AMP-binding_C (PF13193) domains were employed as queries for screening protein sequences containing these specific domains. After screening and analysis, we identified a total of 110 *Ta4CL* genes and renamed them according to their chromosomal positions to enhance the convenience of subsequent research (Table S1). The physicochemical properties of Ta4CLs were evaluated using the ExPASy-ProtParam tool (Table 1). The Ta4CL proteins possess amino acid sequences of varying lengths, ranging from 325 to 1198 residues. These proteins display a wide range of molecular weights, spanning from 35.10 to 130.91 kDa, and theoretical isoelectric points that vary from 5.23 to 9.3. The range of the instability index spans from 23.98 to 49.26, with 70 proteins exhibiting values below 40 and 40 proteins displaying values above 40. This suggests a diminished level of stability within the latter group of proteins. The aliphatic index ranges from 78.19 to 104.94, indicating the varying thermal stability of different Ta4CL proteins. The grand average hydropathy (GRAVY) index spans from −0.232 to 0.309, indicating the degree of hydrophobicity or hydrophilicity. These results offer a comprehensive overview of the entire *Ta4CL* gene family, furnishing vital foundational information for further exploration and examination.

**Figure 1:**
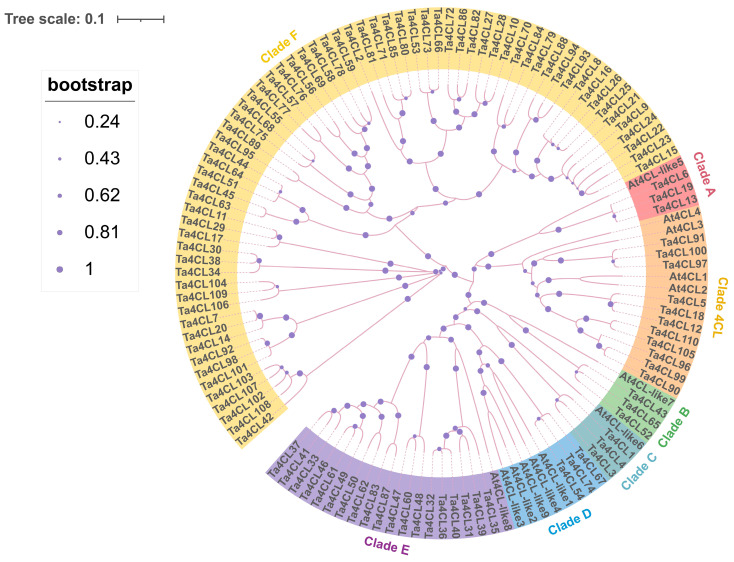
Phylogenetic analysis was conducted to examine the evolutionary relationships between *4CL* genes from *Triticum aestivum* (Ta) and *Arabidopsis thaliana* (At). The resulting phylogenetic tree reveals the evolutionary divergence and relationships within the *4CL* gene family members of these two species. The analysis classified all *4CL* genes into seven distinct clades, with each clade represented by a different color for clear visualization.

To further analyze the gene functions and phylogenetic relationships of the wheat *4CL* family, a clustering analysis was conducted on the homologous genes of *4CL* in wheat and *Arabidopsis* (Figure 1). Subsequently, the evolutionary relationships were classified based on the method for classifying the *4CL* gene family described by *De Azevedo Souza*. The members of the *4CL* gene family were classified into seven distinct clades, denoted as Clade 4CL and A–F. The wheat *4CL* gene located in the Clade 4CL branch consists of a total of 11 genes. Among them, three wheat *4CL* genes, namely, *Ta4CL91*, *Ta4CL97*, and *Ta4CL100*, show close homology to the *At4CL3* gene in *Arabidopsis*. The remaining eight genes, named *Ta4CL5*, *Ta4CL12*, *Ta4CL18*, *Ta4CL90*, *Ta4CL96*, *Ta4CL99*, *Ta4CL105*, and *Ta4CL110*, exhibit relatively close homology to *At4CL1* and *At4CL2* in *Arabidopsis*. These genes are clustered within the 4CL clade, indicating their likely involvement in the biosynthesis of lignin and flavonoids. The Clade A–E encompasses the *Arabidopsis* At4CL-like acyl-CoA synthetase (ACS) proteins. Specifically, Ta4CL6, Ta4CL13, and Ta4CL19 cluster into Clade A, while Ta4CL43, Ta4CL52, and Ta4CL65 cluster into Clade B. Meanwhile, Ta4CL1, Ta4CL3, and Ta4CL4 form a cluster within Clade C, while Ta4CL54, Ta4CL67, and Ta4CL74 are grouped together in Clade D. Additionally, there are 19 Ta4CLs in Clade E branch, demonstrating a relatively large quantity. In Clade F, *At4CL-like* genes are notably absent, with only *Ta4CL* genes forming a distinct branch. This clade is potentially composed of AAEs (acyl-activating enzymes) or AAEL (acyl-activating enzyme-like) proteins.

### 2.2. Chromosomal Distribution and Collinearity Analysis of Ta4CL Genes

**Figure 2:**
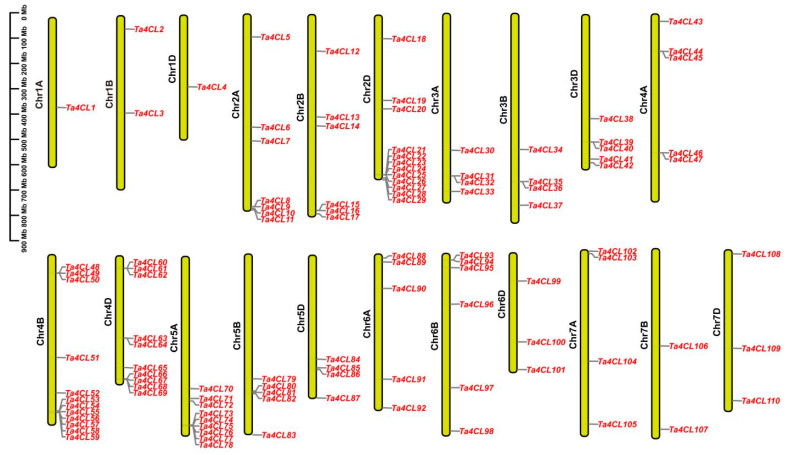
Chromosomal distribution of the *4CL* gene family in wheat. Yellow lines represent chromosomes, while grey lines mark the precise locations of *Ta4CL* genes, which are labeled in red font for emphasis.

Through an analysis of chromosomal distribution, a total of 110 *4CLs* were found to be unevenly mapped across 21 chromosomes from the wheat A, B, and D genomes (Figure 2). Among them, Chromosome 4B and 2D both possess 12 *Ta4CL* genes, exhibiting the highest *Ta4CL* density, at 10.9%. In contrast, Chromosome 1A and 1D each only have one *Ta4CL* gene, showing the lowest *Ta4CL* density, at only 0.9%. In the sub-genomes A, B, and D, there are 35, 37, and 38 members of the *Ta4CL* gene family in each sub-genome, respectively. This represents 31.8%, 33.6%, and 34.5% of the total, showcasing a balanced distribution across the different sub-genomes.

**Figure 3:**
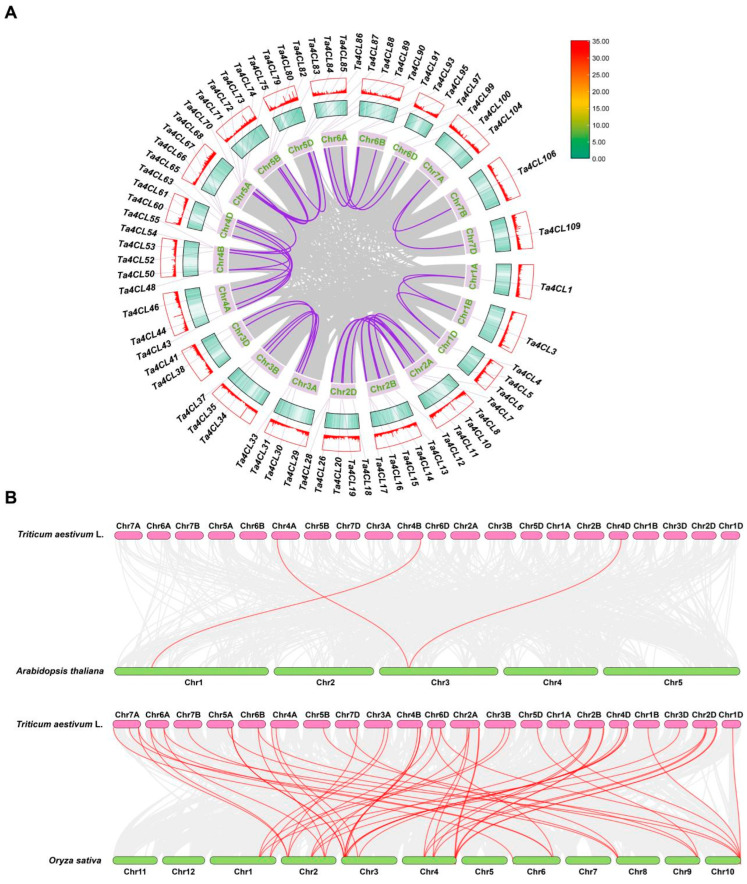
Synteny analysis of *4CL* genes. (**A**) Intraspecific synteny analysis of wheat *4CL* genes. Chromosomes are represented by light-purple boxes, with gray lines connecting collinear gene pairs. The syntenic relationships between wheat *4CL* genes are indicated by dark-purple lines. (**B**) Interspecific synteny of *4CL* genes across various species. The upper and lower collinearity maps illustrate the syntenic relationships between wheat and *Arabidopsis* genes and between wheat and rice genes, respectively. Gray lines connect collinear gene pairs, while red lines specifically highlight collinear *4CL* gene pairs.

An analysis of intraspecific collinearity revealed the presence of 46 pairs exhibiting genomic synteny among 71 genes within the wheat *4CL* gene family (Figure 3A). In the Clade 4CL branch, *Ta4CL91* on Chr6A is collinear with *Ta4CL97* on Chr6B and *Ta4CL100* on Chr6D, and *Ta4CL5* on Chr2A is colinear with *Ta4CL12* on Chr2B and *Ta4CL18* on Chr2D. Additionally, there is collinearity between *Ta4CL90* on Chr6A and *Ta4CL99* on Chr6D. This indicates that whole-genome duplication or polyploidization played a crucial role in driving the evolution of the wheat *4CL* gene family. Subsequently, we conducted collinearity analyses of wheat and *Arabidopsis*, as well as wheat and rice, to investigate the potential evolutionary processes that have shaped *4CLs* (Figure 3B). The results showed that there were 3 collinear gene pairs formed by 3 wheat *4CL* genes and two *Arabidopsis 4CL* genes as well as 57 collinear gene pairs formed by a total of 48 wheat *4CL* genes and 19 rice *4CL* genes. This indicates that the *4CL* family in wheat and rice exhibits a remarkably high level of homology. It is possible that these syntenic gene pairs of *4CL* may possess analogous biological functions or even share a common ancestor.

### 2.3. Analysis of Conserved Motifs, Domains, and Gene Structures Within the 4CL Gene Family in Wheat

**Figure 4:**
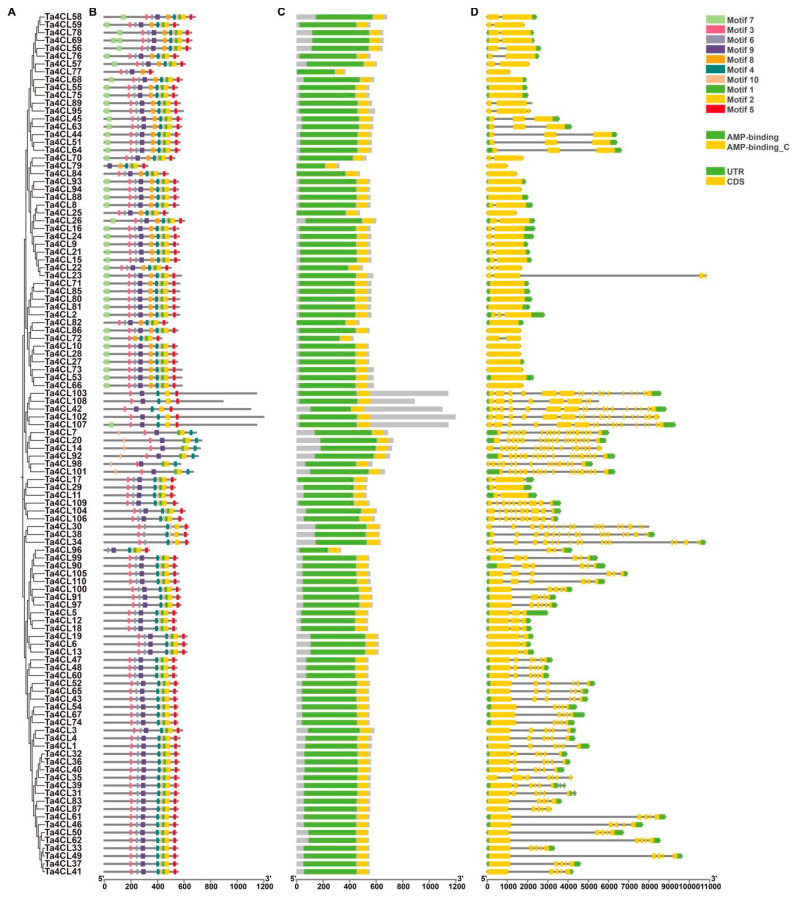
Structural and evolutionary analysis of the *4CL* gene family in wheat. (**A**) Phylogenetic analysis of *4CL* gene family in wheat. The evolutionary tree was constructed using the Neighbor-Joining (NJ) method in MEGA7. (**B**) Conserved protein motifs of Ta4CLs. The motif composition was identified using MEME suite and visualized with TBtools(v2.210), where distinctively colored boxes represent conserved motifs (1–10) with specific amino acid sequences. (**C**) Protein domain architecture of Ta4CLs. Two characteristic domains (AMP-binding and AMP-binding_C) were identified using Pfam and visualized with TBtools, with the AMP-binding domain shown in green and the AMP-binding_C domain in yellow. (**D**) Genomic structures of *Ta4CL* genes. Gene structures were analyzed based on wheat genome annotations and visualized using TBtools, wherein untranslated regions (UTRs) are represented by green boxes, coding sequences (CDSs) are denoted by yellow boxes, and introns are denoted by gray lines.

To gain a more profound understanding of the members of the Ta4CL gene family, we undertook an extensive investigation into the conserved motifs, domains, and gene structures of the identified *Ta4CL* genes (Figure 4). By utilizing MEME, we identified a total of ten distinct motifs within the *Ta4CL* gene family, which were designated as motif 1 through motif 10 (Table S2). The conservative motif abundance of Ta4CL proteins ranged from 5 to 10. Within the same branch, wheat 4CLs shared a similar composition of conserved motifs, highlighting their close evolutionary relationship (Figure 4B). All members of the *Ta4CL* gene family possess the 4CL domain and AFD_class_I superfamily domain in the NCBI CDD database. Additionally, they also feature the AMP binding domain and AMP binding_C domain from the Pfam database (Figure 4C). The number of exons in the *Ta4CL* genes ranges from 1 to 18, and the identified structures of the *Ta4CL* genes exhibit a similar intron–exon distribution within the same clustering branch (Figure 4D).

### 2.4. Analysis of Cis-Acting Elements for Ta4CLs

**Figure 5:**
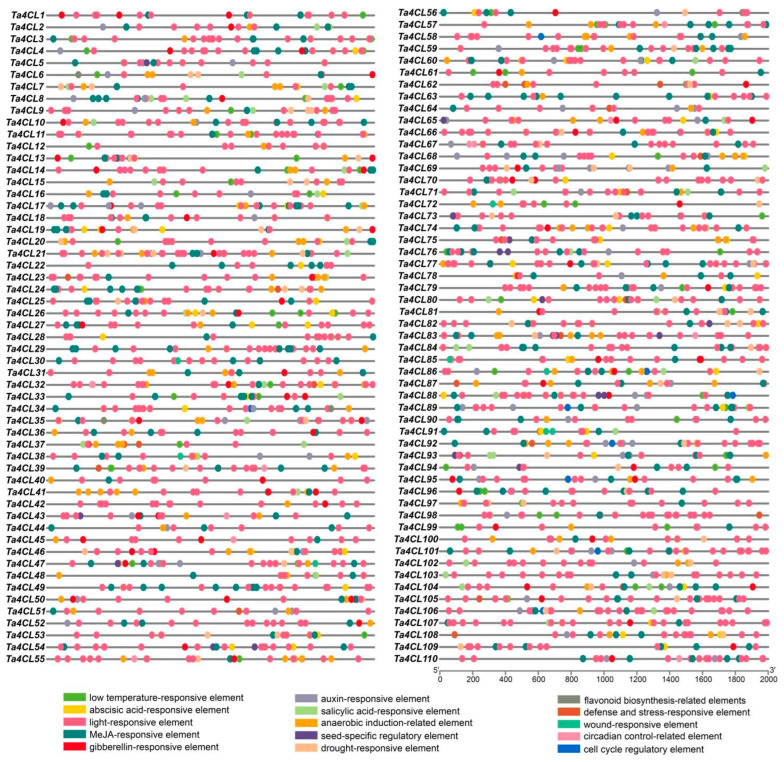
Identification and analysis of cis-regulatory elements in *Ta4CL* gene promoters. The distribution pattern of cis-regulatory elements within the promoter regions of *Ta4CL* genes was systematically analyzed. This schematic representation illustrates the organization of various cis elements, with each distinctly colored ellipse denoting a different type of regulatory element. The promoter regions of individual *Ta4CL* genes are depicted as horizontal grey lines, providing a clear spatial reference for the positioned cis elements.

Cis-acting elements play a crucial role in the transcriptional regulation of genes. Therefore, to further analyze the potential functions of *Ta4CLs*, we predicted the cis-acting elements in the upstream 2000 bp promoter sequences of each member (Figure 5). The promoter regions of the wheat *4CL* gene family are complex and diverse, encompassing a variety of cis-acting elements. These elements are involved in the response to abiotic stresses such as drought and low temperatures as well as plant hormones like salicylic acid, abscisic acid, auxin, methyl jasmonate, and gibberellins. Additionally, this region also includes response elements related to growth and development, including flavonoid biosynthesis, circadian rhythm regulation, and cell cycle control. Together, these functional elements participate in the formation of a sophisticated regulatory mechanism for the wheat *4CL* gene family, providing crucial support for adaptive changes in different environmental conditions. Among the confirmed members of *4CL*, a multitude of promoter regions contain cis-acting elements related to ABA hormone and drought stress response. This indicates that *Ta4CLs* play a crucial role in regulating plant abiotic stress.

### 2.5. Analysis of the Tissue Expression Patterns of Ta4CL Genes

**Figure 6:**
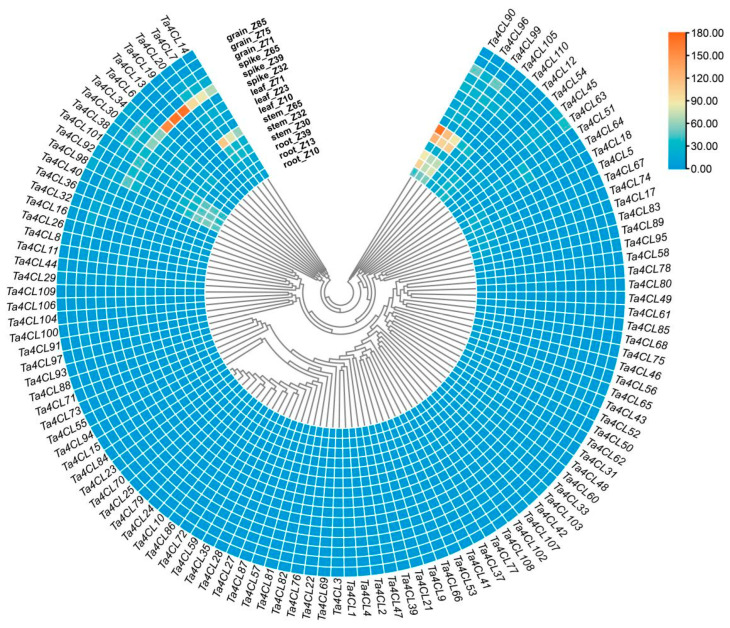
Expression profiling of *Ta4CL* genes across wheat tissues. The transcriptional profiles of *Ta4CL* genes were analyzed across five distinct wheat tissues (spike, root, leaf, grain, and stem tissues) utilizing publicly available RNA-seq data. The developmental stages of wheat samples were precisely determined according to the Zadoks growth scale within the database. To visualize the expression patterns, we generated a heatmap using TBtools(v2.210) software, with gene expression levels quantified as TPM (transcripts per kilobase of exon model per million mapped reads) values.

The expression profiles observed across diverse tissues offer valuable insights into the potential biological roles of genes that warrant further investigation. Therefore, we analyzed the transcriptional levels of the *Ta4CL* genes in five different wheat tissues (spikes, roots, leaves, grains, and stems) using publicly available RNA-seq data (Figure 6). The developmental stages of wheat were assessed based on the Zadoks growth scale within this database. The results show that *Ta4CL6*, *Ta4CL7*, *Ta4CL13*, *Ta4CL19*, and *Ta4CL20* exhibit higher transcription levels in spikes. In stems, *Ta4CL7*, *Ta4CL20*, *Ta4CL90*, *Ta4CL96*, and *Ta4CL99* exhibit elevated expression levels. Additionally, in roots, there are also high transcription levels of *Ta4CL90*, *Ta4CL96*, and *Ta4CL99*. These genes are likely to assume a pivotal role within these specific tissues. The intricate and distinctive tissue-specific expression profiles exhibited by the *Ta4CL* genes underscore their biological functional diversity as well as their potential contributions to the developmental and physiological processes of wheat.

### 2.6. Expression Analysis of Ta4CLs Under Drought and Salt Stress

**Figure 7:**
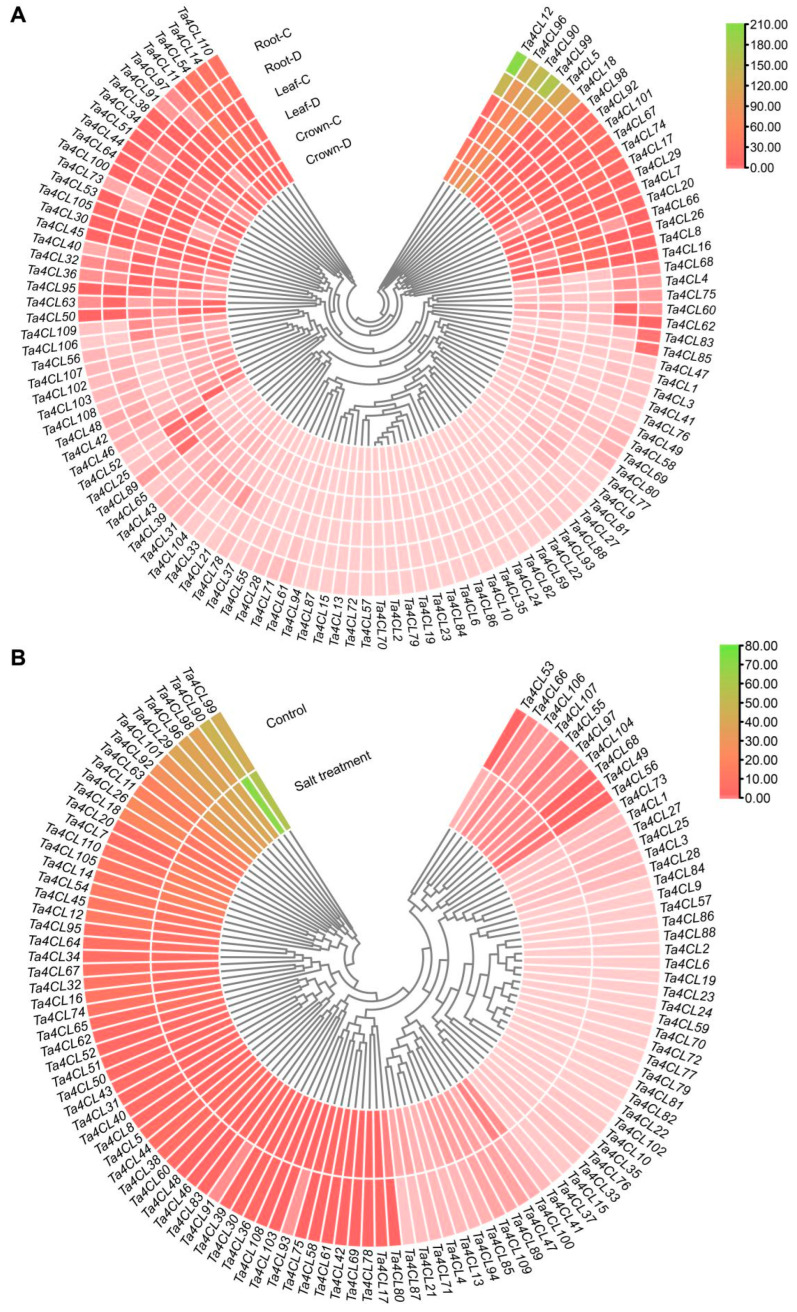
Expression profiling of *Ta4CL* genes under drought and salt stress conditions. (**A**) Differential expression analysis of *Ta4CL* genes in various wheat tissues subjected to drought stress. C, control; D, drought. (**B**) Expression profiles of *Ta4CL* genes under salt stress conditions. The heatmap illustrates differential gene expression patterns, with color gradients representing varying expression levels, as indicated in the color scale bar on the right.

To investigate the response of *Ta4CL* genes to abiotic stress in wheat, we conducted an analysis of the transcriptional changes of *Ta4CL* genes under drought and salt stress conditions using RNA-seq data (NCBI accession number: SRP098756, SRP158842). The results showed that different *Ta4CL* genes exhibit varying degrees of response to drought (Figure 7A) and salt stress (Figure 7B). Under drought stress, the transcription of *Ta4CL45* in wheat crowns and roots exhibited a twofold increase. Additionally, both *Ta4CL53* and *Ta4CL66* showed over-twofold upregulation in crowns and leaves. Furthermore, the transcription levels of *Ta4CL63* and *Ta4CL91* in roots also increased by more than twofold. In contrast, under salinity stress, the transcription levels of *Ta4CL7*, *Ta4CL20*, *Ta4CL83*, *Ta4CL91*, and *Ta4CL93* exhibited an impressive, over-twofold upregulation. It is noteworthy that the *Ta4CL91* gene, located within the Clade 4CL branch, exhibited a more than twofold increase in transcription levels under both salt and drought stress conditions. This observation suggests that this gene may play an increasingly significant role in the plant’s response to abiotic stresses. The varied expression profiles exhibited by the *Ta4CL* genes in response to salinity and drought stress underscore their potential as pivotal regulators in this plant’s response to abiotic challenges.

**Figure 8:**
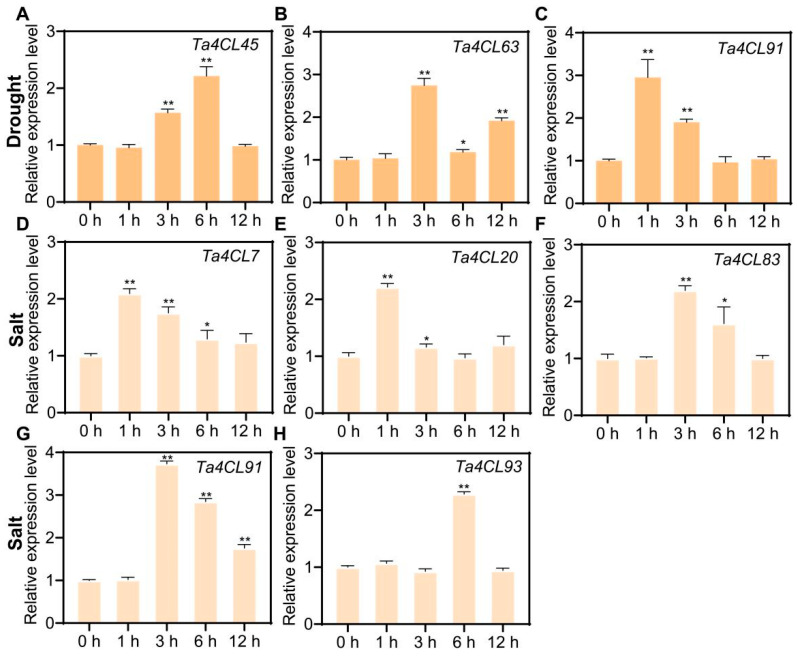
Transcriptional modulation of selected *Ta4CL* genes under salinity and drought stress conditions. (**A**–**C**) Temporal expression patterns of *Ta4CL45* (**A**), *Ta4CL63* (**B**), and *Ta4CL91* (**C**) genes in response to drought stress were monitored 0, 1, 3, 6, and 12 h post-treatment using RT-qPCR. (**D**–**H**) Similarly, the expression profiles of *Ta4CL7* (**D**), *Ta4CL20* (**E**), *Ta4CL83* (**F**), *Ta4CL91* (**G**), and *Ta4CL93* (**H**) genes were analyzed under salt stress conditions at identical time points through RT-qPCR. All data are presented as means ± standard deviations (SDs) from three independent replicates. Statistical analyses were performed using Student’s *t*-test, with asterisks denoting significant differences (* *p* < 0.05, ** *p* < 0.01).

To further validate the drought- and salt-stress-responsive *Ta4CL* genes (showing >2-fold upregulation) identified through transcriptome analysis, we conducted RT-qPCR experiments. The results revealed distinct expression patterns among the *Ta4CL* genes under various stress conditions (Figure 8). Specifically, the transcription levels of *Ta4CL45*, *Ta4CL63*, and *Ta4CL91* were significantly upregulated following drought treatment (Figure 8A–C). Similarly, under salt stress conditions, we observed an increase in transcript abundance for *Ta4CL7*, *Ta4CL20*, *Ta4CL83*, *Ta4CL91*, and *Ta4CL93* (Figure 8D–H). Notably, the consistent upregulation of *Ta4CL91* under both salt and drought treatments highlights its potential critical role in mediating wheat’s response to abiotic stresses.

### 2.7. Ta4CL91 Silencing Compromises Drought and Salt Stress Tolerance in Wheat Seedlings

**Figure 9:**
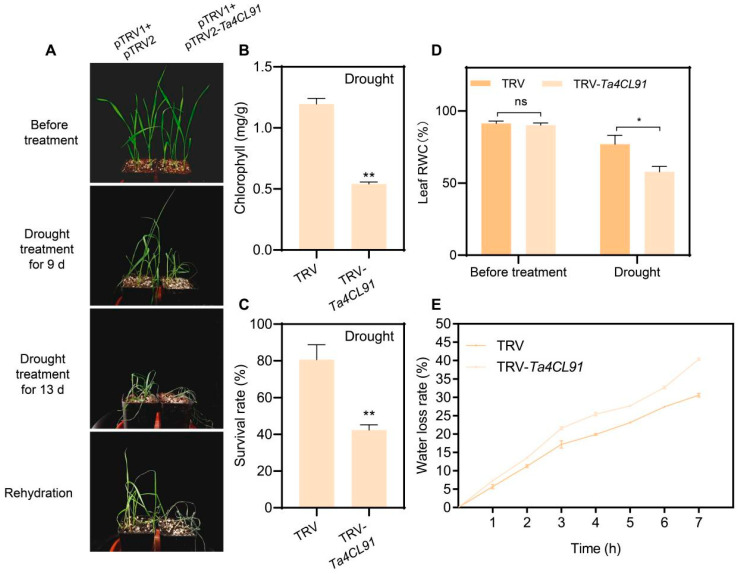
Drought tolerance analysis of *Ta4CL91*-silenced wheat plants. (**A**) Phenotypic comparison between control (CK) and TRV-*Ta4CL91* (VIGS) plants at 9 and 13 days of drought treatment, followed by rewatering. (**B**) Chlorophyll content and (**C**) survival rate measurements for control and TRV-*Ta4CL91* plants after 13 days of drought stress and subsequent rewatering. (**D**) Leaf RWC for control and TRV-*Ta4CL91* plants under well-watered conditions and following drought stress. (**E**) Leaf water loss rates for detached leaves from control and TRV-*Ta4CL91* plants. Data were analyzed using Student’s *t*-test, with statistical significance denoted as * *p* < 0.05 and ** *p* < 0.01.

*Ta4CL91*‘s activity is induced by both drought and salt stress, and it is phylogenetically classified within the 4CL clade. To investigate the functional role of *Ta4CL91* in wheat under abiotic stress conditions, we employed virus-induced gene silencing using the TRV vector system to specifically downregulate *Ta4CL91* expression (Figure S1). For VIGS validation, we included a positive control by targeting the *TaPDS* gene, which resulted in characteristic albino phenotypes in wheat seedlings following TRV-*TaPDS* inoculation (Figure S2). Phenotypic analysis demonstrated that the TRV-*Ta4CL91* plants exhibited significantly reduced drought tolerance compared to the control lines after 9 and 13 days of drought stress (Figure 9A). This was evidenced by marked decreases in chlorophyll content (Figure 9B), survival rates (Figure 9C), and leaf relative water content (Figure 9D), accompanied by an increased rate of water loss in detached leaves (Figure 9E).

**Figure 10:**
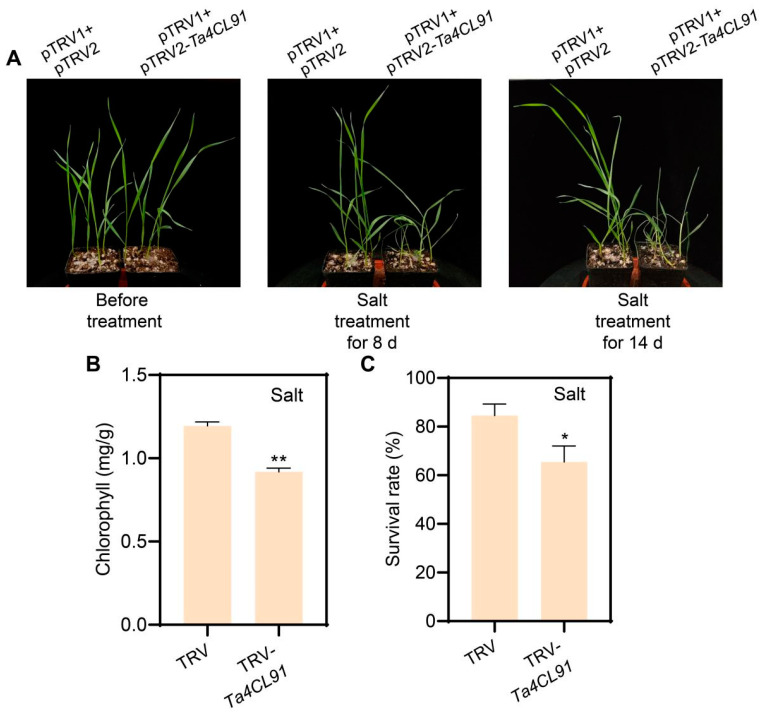
Salt tolerance analysis of *Ta4CL91*-silenced wheat plants. (**A**) Phenotypic responses of control and TRV-*Ta4CL91* plants to salt stress at 8 and 14 days post-treatment. (**B**) Chlorophyll content and (**C**) survival rate of control and TRV-*Ta4CL91* plants following salt stress exposure. Statistical analyses were performed using Student’s *t*-test, with significance levels indicated as * *p* < 0.05 and ** *p* < 0.01.

Similarly, following 8 days of salt stress treatment, the TRV-*Ta4CL91* plants displayed compromised salt tolerance relative to the control plants (Figure 10A). The phenotypic differences became increasingly pronounced by the 14th day of salt treatment (Figure 10A), with statistically significant reductions in both chlorophyll content (Figure 10B) and survival rate (Figure 10C). These findings strongly suggest that *Ta4CL91* plays a crucial role in mediating plant responses to both drought and salt stress conditions.

## 3. Discussion

The members of the *4CL* gene family have been documented to play a pivotal role not only in the intricate processes of plant growth and development but also in mediating responses to both biotic and abiotic stresses [5,25,26]. Elevated 4CL activity enhances lodging resistance in wheat stems [27]. Compared to low-density (LD) homogeneous distribution, high-density (HD) treatment significantly reduced 4CL activity and *Ta4CL* gene expression in culms during their critical formation period [28]. The *4CL* genes possess the ability to encode a variety of enzymes and exhibit distinct substrate affinities, which seem to correspond to diverse metabolic functions [5,10]. However, there exists a notable lack of comprehensive analyses regarding the *4CL* genes within the realm of wheat. In this study, we conducted a comprehensive identification of 110 *Ta4CL* genes at the whole-genome level (Table 1) and performed an in-depth analysis of their fundamental characteristics (Figure 1, Figure 2, Figure 3, Figure 4 and Figure 5). Furthermore, we examined the expression patterns of these genes across various tissues (Figure 6) and investigated the transcriptional alterations under drought and salt stress conditions (Figure 7 and Figure 8). The aim was to identify *Ta4CL* genes with potential for stress resistance, providing important target genes for breeding wheat varieties that are tolerant to salt and drought. As performed by *De Azevedo Souza* [10], the members of the *4CL* gene family in wheat were categorized into seven distinct clades, designated as Clade 4CL and A-F (Figure 1). The diverse isoenzymes of 4CL exhibit distinct substrate utilization preferences, indicating that the genes and enzymes associated with 4CL have undergone a process of subfunctionalization tailored for the biosynthesis of various classes of phenylpropanoid-derived compounds [5]. At4CL1–4 is situated within the clade of the 4CL branch and subsequently diverges into two distinct types. At4CL1, At4CL2, and At4CL4 are classified under type I, which is intimately linked to lignin biosynthesis, whereas At4CL3 resides within the type II cluster, associated with the biosynthesis of phenylpropanoids other than lignin [11,12]. In the clade of 4CL, eight genes demonstrate a relatively high degree of homology with *At4CL1* and *At4CL2* found in *Arabidopsis* (Figure 1). These *4CL* genes are likely implicated in lignin biosynthesis, while *Ta4CL91*, *Ta4CL97*, and *Ta4CL100* show close homology to the *At4CL3* gene in *Arabidopsis*, suggesting their potential involvement in the biosynthesis of phenylpropanoids other than lignin (Figure 1). The Ta4CLs situated within Clade A-E exhibit a close homologous relationship with the At4CL-like ACS proteins (Figure 1), suggesting that these genes may not encode enzymes possessing 4CL activity and are devoid of functionality towards the known hydroxycinnamate substrates associated with 4CL. Based on the previously reported literature [10,29,30], the Ta4CLs within Clade F (Figure 1) may comprise either AAE or AAEL proteins.

Proteins’ physicochemical properties play a key role in plant stress resistance. For instance, heat shock proteins (HSPs) are highly conserved across species, maintaining proteostasis and protecting cells under stress [31,32]. Similarly, late embryogenesis abundant (LEA) proteins are small, hydrophilic polypeptides (10–30 kDa) with heat stability and diverse protective functions, including antioxidant activity, ion binding, and macromolecule stabilization [33]. In this study, different Ta4CL proteins exhibited distinct physicochemical properties (Table 1), likely influencing their stress tolerance capacities. The structural characteristics of genes, along with the conserved motifs and domains of their corresponding proteins, significantly influence gene functionality [34]. The Ta4CL proteins residing within the same phylogenetic branch exhibit a remarkable degree of similarity in both the types and quantities of conserved motifs (Figure 4B). Furthermore, the exon–intron architecture of these corresponding genes also reveals notable parallels (Figure 4D), underscoring the functional conservation among *Ta4CLs* clustered within identical branches. *Ta4CLs* are distributed across all chromosomes in both gene clusters and dispersed forms (Figure 2). Within wheat, there are 46 collinear gene pairs associated with *Ta4CLs* (Figure 3A). Polyploidization, segmental duplication, and tandem duplication events may have driven the evolution of the *Ta4CL* gene family. Notably, there are only 3 gene pairs between wheat and *Arabidopsis 4CLs*, whereas there are 57 gene pairs between wheat and rice (Figure 3B), further indicating the conservation of *4CLs* among species within the Poaceae family.

Wheat encounters a myriad of abiotic stresses throughout its growth and development, with drought and salinity consistently posing significant challenges during its reproductive phase [35,36]. Numerous studies have demonstrated that the *4CL* gene plays a crucial role in responding to and regulating the processes associated with both drought and salt stress [21,22,24,37]. However, a comprehensive analysis of the functions of *4CLs* in wheat’s response to these adversities remains conspicuously underexplored. The aim of analyzing cis-acting elements in gene promoter regions is to predict their potential involvement in biological processes [38]. In this study, a significant proportion of the identified *Ta4CL* genes were found to contain cis-acting elements that respond to the abscisic acid (ABA) hormone and drought conditions (Figure 5). Previous studies have demonstrated that ABA plays a critical role in mediating plant responses to various abiotic stresses [39,40]. The presence of these ABA-responsive elements in *Ta4CLs* strongly suggests their essential function in conferring abiotic stress tolerance, highlighting their potential importance in plant stress adaptation mechanisms. To elucidate the functional relevance of *Ta4CL* genes under abiotic stress conditions, we systematically analyzed their transcriptional dynamics using RNA-seq data under drought and salt stress treatments (Figure 7). Distinct expression patterns were observed among *Ta4CL* family members, with individual genes demonstrating stress-specific regulation. This differential transcriptional responsiveness highlights their specialized roles in mediating plants’ adaptation to environmental stresses. Transcriptomic analysis and RT-qPCR validation under drought and salt stress revealed that *Ta4CL45*, *Ta4CL63*, and *Ta4CL91* were significantly upregulated under drought conditions, whereas *Ta4CL7*, *Ta4CL20*, *Ta4CL83*, *Ta4CL91*, and *Ta4CL93* showed increased expression under salt stress (Figure 7 and Figure 8). Notably, *Ta4CL91* showed responsiveness to both salinity and drought stress conditions (Figure 7 and Figure 8), indicating its essential regulatory role in mediating wheat’s adaptive responses to abiotic stress. To functionally characterize *Ta4CL91* in stress responses, we employed VIGS [41] to knock down its expression in wheat seedlings (Figure S1). Phenotypic analysis revealed that *Ta4CL91*-silenced plants displayed enhanced sensitivity to both salt and drought stress conditions compared to that of the controls (Figure 9 and Figure 10). These findings collectively demonstrate that *Ta4CL91* serves as a key regulatory component in the molecular network governing wheat’s adaptation to abiotic stress. In crop breeding, the introduction of stress-resistance genes frequently improves stress tolerance but typically incurs growth penalties or yield reduction. This trade-off primarily stems from the substantial ATP and carbon resources consumed by stress-response pathways, which are consequently diverted from reproductive growth [42]. Moreover, constitutive activation of stress-signaling pathways may suppress developmental genes, as exemplified by *DREB1A* overexpression; while enhancing drought resistance, it simultaneously leads to stunted growth [43]. Therefore, achieving an optimal balance between stress resistance and agronomic performance remains a critical challenge in modern crop improvement.

## 4. Materials and Methods

### 4.1. Genome-Wide Identification of Members of the 4CL Gene Family in Wheat

Two hidden Markov models corresponding to the AMP-binding (PF00501) and AMP-binding_C (PF13193) domains were retrieved from the Pfam database (http://pfam-legacy.xfam.org/, accessed on 18 April 2024) [44]. These models were subsequently utilized as queries to screen the protein sequences of Ta4CL. Following this, both the Pfam and NCBI Conserved Domains Datebase (CDD) (https://www.ncbi.nlm.nih.gov/Structure/bwrpsb/bwrpsb.cgi, accessed on 18 April 2024) were employed to verify whether the selected Ta4CL proteins contain the AMP-binding domain and AMP-binding_C domain, thereby enabling us to identify members of the *Ta4CL* gene family.

### 4.2. Phylogenetic Analysis and Physicochemical Property Assessment

The amino acid sequences of the 4CL proteins from wheat and *Arabidopsis thaliana* were obtained from Ensembl Plants (https://plants.ensembl.org/index.html, accessed on 8 May 2024). Multiple sequence alignment was performed using MEGA7 (https://www.megasoftware.net/, accessed on 8 May 2024) [45], and phylogenetic tree construction was conducted employing the Neighbor-Joining (NJ) method. The phylogenetic tree was refined using the iTOL online tool (https://itol.embl.de/, accessed on 28 May 2024). The ProtParam online tool available on ExPASy (https://web.expasy.org/protparam/, accessed on 18 July 2024) [46] was utilized to predict the physicochemical properties of Ta4CL proteins. This analysis primarily encompasses a variety of parameters, including amino acid numbers, molecular weight of the protein, instability index, theoretical isoelectric point, and the grand average of hydropathicity (GRAVY), among others.

### 4.3. Analysis of Chromosomal Distribution and Gene Collinearity

The distribution of the *4CL* gene family in wheat chromosomes was determined based on the annotation file of the wheat genome, and the physical distribution of candidate *Ta4CL* genes on wheat chromosomes was annotated using TBtools(v2.210) [47]. To facilitate further research, the wheat *4CL* genes were renumbered according to their chromosomal positions. TBtools(v2.210) was utilized to analyze the gene collinearity relationships within wheat species as well as between wheat and *Arabidopsis* and between wheat and rice across different species. The results were visualized using TBtools(v2.210); subsequently, modifications were made, and the saved SVG-format images were integrated through Adobe Illustrator(v24.2.1) software.

### 4.4. Analysis of Conserved Motifs, Domains, and Gene Structure

The phylogenetic tree in Newick format was derived from MEGA7 (https://www.megasoftware.net/, accessed on 8 May 2024) [45]. The conserved motifs were analyzed using the MEME Suite version 5.5.5 (https://meme-suite.org/meme/tools/meme, accessed on 18 July 2024) [48] online platform, resulting in the generation of a meme.xml file. For the analysis of conserved domains, we employed the batch CD-search tool [49] provided by NCBI (https://www.ncbi.nlm.nih.gov/Structure/bwrpsb/bwrpsb.cgi, accessed on 18 July 2024), which yielded a hitdata.txt file. Gene structures were represented using wheat genome annotation files. Some of these files underwent modifications; ultimately, all files were imported into TBtools(v2.210) for comprehensive visualization.

### 4.5. Analysis of Cis-Acting Elements in Promoter Regions

The 2000 bp upstream promoter regions of *Ta4CL* genes, adjacent to the start codon of the coding sequence, were subjected to cis-acting element analysis using PlantCARE (http://bioinformatics.psb.ugent.be/webtools/plantcare/html/, accessed on 19 July 2024) [50]. After modifying the output files from PlantCARE, visualization was performed using TBtools(v2.210). The generated SVG format image was subsequently refined and enhanced with Adobe Illustrator software.

### 4.6. Expression Analysis Involving Varying Tissues Under Drought and Salinity Stress

The expression data regarding the *Ta4CL* genes in various tissues were derived from five distinct wheat tissues—spike, root, leaf, grain, and stem tissue—utilizing publicly accessible RNA-seq datasets (http://202.194.139.32/expression/wheat.html, accessed on 30 July 2024) [51]. Furthermore, the expression profiles of *Ta4CL* genes under drought and salt stress conditions were sourced from SRA data (SRP098756, SRP158842). The TPM (transcripts per kilobase of exon model per million mapped reads) and FPKM (fragments per kilobase of exon model per million mapped fragments) values for the *Ta4CL* genes were employed to construct a heat map using TBtools(v2.210).

### 4.7. Analysis of Drought and Salt Stress Responses in Selected Ta4CL Genes

To investigate the stress-responsive expression patterns of selected *Ta4CL* genes, a time-course experiment was conducted using 12-day-old Jimai22 wheat seedlings. The plants were exposed to drought stress and salt stress conditions through treatment with 5% polyethylene glycol 6000 (Sigma-Aldrich, St. Louis, MO, USA) and 200 mM NaCl solution, respectively. Root tissue samples were collected at five distinct time points (0, 1, 3, 6, and 12 h) following stress initiation to monitor the temporal dynamics of gene expression. Three independent biological replicates were established per treatment condition, with each replicate comprising a composite sample from three individual plant lines. Immediately after collection, all samples were flash-frozen in liquid nitrogen to preserve RNA integrity and stored at −80 °C until further molecular analysis.

### 4.8. RNA Isolation, Reverse Transcription, and qPCR Analysis

Total RNA was isolated from the samples using the RNAiso Easy Kit (TaKaRa, Kyoto, Japan) according to the manufacturer’s protocol. To ensure genomic DNA elimination and subsequent cDNA synthesis, we employed the PrimeScript™ FAST RT Reagent Kit with gDNA Eraser (TaKaRa, Japan). Quantitative real-time PCR (RT-qPCR) analysis was then conducted using the 2× SYBR Green qPCR Master Mix (Selleck, Houston, TX, USA) on the LineGene9600 fluorescence quantitative PCR system (Bioer Technology, Hangzhou, China). Gene expression quantification was performed using the 2^−ΔΔCt^ method [52], with the wheat *Actin* gene serving as an internal control. The sequence-specific primers used for RT-qPCR amplification were carefully designed, and their details are provided in Table S3.

### 4.9. VIGS Vector Construction and Infection Methodology

The virus-induced gene-silencing system was established using pTRV1 and pTRV2 vectors obtained from Fenghui Biotechnology Co., Ltd. (Changsha, China). To design optimal VIGS target fragments, we employed the pssRNAit computational platform (https://www.zhaolab.org/pssRNAit/, accessed on 28 September 2024), an advanced bioinformatics tool specifically developed for plant small-RNA analysis and efficient VIGS fragment prediction. Based on the computational analysis, we selected two distinct gene-specific fragments: a 192 bp sequence from the *Ta4CL91* coding region and a 188 bp sequence from the *TaPDS* coding region. These fragments were subsequently amplified and directionally cloned into the pTRV2 vector through restriction-enzyme-mediated cloning. Specifically, the insertion was performed using the *Eco*RI and *Bam*HI restriction sites, which facilitated the generation of the recombinant constructs pTRV2-*Ta4CL91* and pTRV2-*TaPDS*, respectively. The successful construction of these recombinant vectors was confirmed through sequencing verification.

For seed sterilization and germination, wheat cultivar Jimai22 seeds were subjected to a surface sterilization protocol. Initially, seeds were treated with 75% (*v*/*v*) ethanol for 1 min to remove surface contaminants; this was followed by immersion in 2.5% sodium hypochlorite solution supplemented with 0.1% Tween 20 for 5 min to ensure surface sterilization. After chemical treatment, seeds were thoroughly washed five times with sterile deionized water to remove residual sterilizing agents. For germination, the sterilized seeds were aseptically transferred onto 2–3 layers of sterile filter paper moistened with distilled water in a controlled-environment chamber. The germination process was carried out at a constant temperature of 28 °C under dark conditions for 30 h to ensure seed germination.

The *Agrobacterium*-mediated genetic transformation was conducted using a well-established VIGS protocol. We employed *Agrobacterium tumefaciens* strain GV3101 containing either the pTRV1 vector or various pTRV2-derived constructs (pTRV2, pTRV2-*Ta4CL91*, and pTRV2-*TaPDS*). Bacterial cultures were grown in Luria–Bertani (LB) medium supplemented with 100 mg·L^−1^ of rifampicin and 50 mg·L^−1^ of kanamycin at 28 °C with constant shaking at 180 rpm until reaching the optimal optical density (OD_600_ = 0.3). For the agroinfiltration process, we prepared a bacterial suspension by mixing equal volumes (1:1 ratio) of pTRV1-containing and pTRV2-derived vector-containing *Agrobacterium* cultures in an optimized infiltration buffer containing 19.62 mg·L^−1^ of acetosyringone (AS), 400 mg·L^−1^ of cysteine (Cys), and 5 mL·L^−1^ of Tween 20 to enhance transformation efficiency. The transformation procedure was conducted utilizing a specialized vacuum infiltration system. Approximately 5 mL of the bacterial suspension was transferred into 10 mL sterile medical glass bottles fitted with rubber caps. Surface-sterilized germinated wheat seeds were then placed in these bottles. Vacuum infiltration was performed for 30 seconds using a 20 mL syringe to ensure efficient entry of bacteria into the plant tissues. Following this vacuum treatment, the seeds were co-cultivated with the *Agrobacterium* suspension in a controlled environment shaker at 28 °C and 180 rpm for a duration of 15 h. After co-cultivation, the agroinfiltrated germinated wheat seeds underwent thorough washing with sterilized water to remove surface-adhered Agrobacteria. The transformed seeds were subsequently transferred to a sterilized soil mixture for further growth and phenotypic analysis. The entire infection procedure adhered to previously optimized protocols [41].

### 4.10. Tolerance Analysis Under Drought and Salt Stress Conditions

For the natural drought treatment, 12-day-old CK and VIGS wheat seedlings underwent water deprivation for 9 and 13 days, with phenotypic observations recorded and photographs taken. After rehydration, survival rates and chlorophyll content were measured. For salt treatment, seedlings were irrigated with 200 mM NaCl and then subjected to phenotypic observations and photography at days 8 and 14. Survival rates and chlorophyll content were also assessed to evaluate stress responses. For each replicate, approximately 20 individual plants were included for both the control and gene-silenced lines per treatment, with three independent replicates performed.

### 4.11. Measurement of Relative Water Content and Water Loss Rate in Detached Leaves

To measure relative water content (RWC), fresh leaves were collected from plants and weighed to determine fresh weight (FW). The leaves were then soaked in distilled water at 25 °C in the dark for 8 h, and their turgid weight (TW) was measured. After oven-drying at 65 °C to constant weight, the leaves’ dry weight (DW) was recorded. RWC was calculated as follows: RWC (%) = [(FW − DW)/(TW − DW)] × 100 [53].

For the water loss assays, leaves from wheat seedlings were promptly weighed and transferred to a growth chamber maintained at ambient temperature. The leaves were then weighed at hourly intervals. The water loss rate (WLR) was calculated using the following formula: WLR (%) = [(Initial fresh weight − Current fresh weight)/Initial fresh weight] × 100 [24].

## 5. Conclusions

In this study, we systematically identified the *4CL* gene family in wheat, revealing both conserved features within phylogenetic clades and variations among individual *Ta4CL* members. Promoter analysis revealed that a substantial number of *Ta4CL* genes possess cis-regulatory elements associated with stress responses, particularly those responsive to ABA and drought stress. Transcriptional profiling demonstrated the responsiveness of multiple *Ta4CL* genes to diverse abiotic stresses. Particularly, *Ta4CL91*, a member of the 4CL clade, showed marked upregulation under both drought and salt stress conditions. Functional characterization through VIGS-mediated silencing revealed that the *Ta4CL91*-deficient plants displayed compromised stress tolerance compared to that of the wild-type controls. These findings not only provide a comprehensive identification of the *4CL* gene family in wheat but also explore the responses and involvement of *Ta4CL* members, particularly *Ta4CL91*, in abiotic stress adaptation.

## Figures and Tables

**Table 1:** Identification and analysis of physicochemical properties of the *4CL* gene family members in wheat.

Gene	Gene ID	No. of Amino Acids	Mol. Wt (Da)	Isoelectric Point (pI)	Instability Index (II)	Aliphatic Index	Grand Average of Hydropathicity (GRAVY)
*Ta4CL1*	TraesCS1A02G196700	568	60,239.3	7.72	43	96.94	0.141
*Ta4CL2*	TraesCS1B02G067600	564	61,444.37	6.17	35.62	89.95	−0.048
*Ta4CL3*	TraesCS1B02G211300	587	62,541.94	8.78	48.46	96.46	0.119
*Ta4CL4*	TraesCS1D02G200200	568	60,358.44	8.54	47.57	96.06	0.13
*Ta4CL5*	TraesCS2A02G145800	542	57,762.92	5.65	33.4	101.11	0.173
*Ta4CL6*	TraesCS2A02G272900	620	66,508.68	6.27	40.33	96.42	0.102
*Ta4CL7*	TraesCS2A02G290900	690	76,141.63	5.64	23.98	80.55	−0.187
*Ta4CL8*	TraesCS2A02G556900	558	60,323.35	6.87	40.81	86.99	−0.024
*Ta4CL9*	TraesCS2A02G557100	557	60,187.48	8.3	35.91	86.62	−0.006
*Ta4CL10*	TraesCS2A02G570500	545	59,224.46	8.56	29.14	96.99	0.036
*Ta4CL11*	TraesCS2A02G581600	529	55,281.21	6.16	27.28	95.69	0.15
*Ta4CL12*	TraesCS2B02G171200	540	57,381.47	5.58	34.13	101.15	0.173
*Ta4CL13*	TraesCS2B02G291100	619	66,374.5	6.21	40.07	96.25	0.106
*Ta4CL14*	TraesCS2B02G307300	720	79,208.5	6.48	32.26	81.29	−0.162
*Ta4CL15*	TraesCS2B02G586300	565	61,427.81	8.03	37.25	86.96	−0.025
*Ta4CL16*	TraesCS2B02G587100	558	60,337.42	7.16	39.98	87.69	−0.029
*Ta4CL17*	TraesCS2B02G605800	536	55,673.58	6.1	25.21	96.27	0.167
*Ta4CL18*	TraesCS2D02G150400	540	57,178.25	5.7	33.88	100.43	0.186
*Ta4CL19*	TraesCS2D02G272200	619	66,428.59	6.17	40.07	96.58	0.11
*Ta4CL20*	TraesCS2D02G288800	731	80,522.71	6.01	29.53	79.53	−0.179
*Ta4CL21*	TraesCS2D02G556000	563	61,094.64	8.72	34.52	87.96	−0.009
*Ta4CL22*	TraesCS2D02G556100	501	54,550.86	9.3	36.53	79.38	−0.105
*Ta4CL23*	TraesCS2D02G556500	578	62,772.43	7.63	38.39	87.37	0.015
*Ta4CL24*	TraesCS2D02G556600	564	61,297.7	8.01	36.13	83.83	−0.028
*Ta4CL25*	TraesCS2D02G556900	478	51,616.28	6.16	40.48	87.28	−0.049
*Ta4CL26*	TraesCS2D02G557900	601	64,808.66	8.23	45.49	87.77	−0.03
*Ta4CL27*	TraesCS2D02G581200	548	59,725.13	8.41	28.24	98.41	0.055
*Ta4CL28*	TraesCS2D02G581900	548	59,739.14	8.56	30.03	97.14	0.04
*Ta4CL29*	TraesCS2D02G598500	531	55,517.3	5.85	27.58	94.97	0.127
*Ta4CL30*	TraesCS3A02G304600	631	69,199.48	7.29	36.07	89.95	−0.143
*Ta4CL31*	TraesCS3A02G394200	558	58,793.98	8.56	48.52	96.58	0.191
*Ta4CL32*	TraesCS3A02G394300	560	58,750.7	7.18	44.01	97.29	0.18
*Ta4CL33*	TraesCS3A02G472200	550	58,403.73	8.77	44.6	97.42	0.177
*Ta4CL34*	TraesCS3B02G331900	634	69,537.89	7.03	38.38	89.35	−0.13
*Ta4CL35*	TraesCS3B02G426200	560	58,750.76	6.44	49.26	94.11	0.213
*Ta4CL36*	TraesCS3B02G426300	562	58,863.77	6.85	43.96	97.63	0.188
*Ta4CL37*	TraesCS3B02G515400	550	58,190.55	8.93	46.19	98.13	0.218
*Ta4CL38*	TraesCS3D02G297300	626	68,744.91	7.63	37.9	88.93	−0.174
*Ta4CL39*	TraesCS3D02G388000	558	58,856.93	7.71	47.81	97.1	0.155
*Ta4CL40*	TraesCS3D02G388100	562	58,981.88	6.85	46.12	96.42	0.164
*Ta4CL41*	TraesCS3D02G468000	554	58,775.37	9.2	45.59	98.12	0.204
*Ta4CL42*	TraesCS3D02G491300	1100	120,520.26	5.82	44.49	86.14	−0.098
*Ta4CL43*	TraesCS4A02G036200	549	58,531.44	6.39	37.99	98.11	0.154
*Ta4CL44*	TraesCS4A02G119100	567	61,510.76	7.59	27.34	89.52	−0.025
*Ta4CL45*	TraesCS4A02G119200	579	63,406.05	7.57	30.9	84.23	−0.092
*Ta4CL46*	TraesCS4A02G239000	551	58,574.87	8.7	43.53	94.92	0.151
*Ta4CL47*	TraesCS4A02G239100	543	57,096.28	8.71	45.96	102.45	0.309
*Ta4CL48*	TraesCS4B02G075900	543	57,570.91	8.16	44.71	104.94	0.308
*Ta4CL49*	TraesCS4B02G076000	551	58,594.8	8.79	43.77	95.81	0.173
*Ta4CL50*	TraesCS4B02G076100	543	57,750.89	8.91	48	99.39	0.179
*Ta4CL51*	TraesCS4B02G185400	567	61,574.8	7.59	27.46	88.48	−0.03
*Ta4CL52*	TraesCS4B02G269200	555	59,004.06	6.48	38.34	98.97	0.184
*Ta4CL53*	TraesCS4B02G323900	581	63,176.8	7.97	32.61	91.98	−0.009
*Ta4CL54*	TraesCS4B02G326800	552	59,349.51	8.59	36.05	96.32	0.052
*Ta4CL55*	TraesCS4B02G329700	549	58,957.74	6.68	36.76	86.03	−0.005
*Ta4CL56*	TraesCS4B02G329800	650	70,840.11	6.9	41.11	85.22	−0.083
*Ta4CL57*	TraesCS4B02G329900	606	66,458.34	7.31	45.75	87.61	−0.081
*Ta4CL58*	TraesCS4B02G330100	682	74,136.93	7.65	38.61	84.28	−0.073
*Ta4CL59*	TraesCS4B02G330200	558	60,755.62	6.42	33.99	84.61	−0.039
*Ta4CL60*	TraesCS4D02G074500	543	57,442.68	8.49	46.4	103.54	0.291
*Ta4CL61*	TraesCS4D02G074600	551	58,505.67	8.56	43.25	95.48	0.156
*Ta4CL62*	TraesCS4D02G074700	546	57,945.91	9.03	48.4	96.56	0.109
*Ta4CL63*	TraesCS4D02G186700	579	63,262.76	7.58	30.1	82.56	−0.107
*Ta4CL64*	TraesCS4D02G186800	567	61,535.77	7.24	27.2	89.35	−0.024
*Ta4CL65*	TraesCS4D02G268400	547	58,328.24	6.53	39.12	98.99	0.181
*Ta4CL66*	TraesCS4D02G320900	583	63,280.96	7.97	33.35	91.85	−0.006
*Ta4CL67*	TraesCS4D02G323500	551	59,036.2	8.86	35.98	96.52	0.062
*Ta4CL68*	TraesCS4D02G326700	585	63,149.89	6.3	38.49	90.43	0.03
*Ta4CL69*	TraesCS4D02G327100	656	70,903.94	7.32	40.32	80.49	−0.127
*Ta4CL70*	TraesCS5A02G307500	528	57,725.39	8.79	40.82	86.95	−0.098
*Ta4CL71*	TraesCS5A02G356800	565	60,791.66	7	27.92	89.63	−0.025
*Ta4CL72*	TraesCS5A02G368300	430	46,523.43	7.66	34.52	93.65	−0.006
*Ta4CL73*	TraesCS5A02G496400	583	63,340.09	7.97	33.31	91.36	−0.005
*Ta4CL74*	TraesCS5A02G498800	552	59,198.41	8.74	37.86	96.34	0.061
*Ta4CL75*	TraesCS5A02G501200	549	58,993.9	6.48	37.54	88.52	0.016
*Ta4CL76*	TraesCS5A02G501300	558	60,574.43	6.66	37.31	84.46	−0.06
*Ta4CL77*	TraesCS5A02G501400	369	40,277.61	7.74	38.7	92.55	0.067
*Ta4CL78*	TraesCS5A02G501700	654	70,995.27	8.03	39.95	84.31	−0.077
*Ta4CL79*	TraesCS5B02G307900	325	35,103.07	7.62	33.36	83.05	−0.141
*Ta4CL80*	TraesCS5B02G359300	565	60,867.83	7.32	27.07	88.41	−0.014
*Ta4CL81*	TraesCS5B02G365000	562	60,813.73	6.61	35.77	92.38	−0.015
*Ta4CL82*	TraesCS5B02G370600	475	51,264.79	5.84	26.99	95.45	0.033
*Ta4CL83*	TraesCS5B02G570100	554	58,737.12	8.54	45.32	100	0.195
*Ta4CL84*	TraesCS5D02G314500	479	52,202.71	5.94	35.01	84.86	−0.128
*Ta4CL85*	TraesCS5D02G365800	565	60,803.71	7.02	27.93	89.45	−0.018
*Ta4CL86*	TraesCS5D02G377800	551	59,564.56	6.74	26.78	98.02	0.068
*Ta4CL87*	TraesCS5D02G561500	555	58,863.23	8.82	47.68	99.08	0.184
*Ta4CL88*	TraesCS6A02G029900	558	60,265.29	5.36	41.18	90.34	0.077
*Ta4CL89*	TraesCS6A02G059400	569	60,521.64	6.81	37.22	86.33	0.078
*Ta4CL90*	TraesCS6A02G151700	546	58,531.58	5.23	35.96	97.73	0.093
*Ta4CL91*	TraesCS6A02G266700	573	60,626.98	5.44	33.6	100.28	0.234
*Ta4CL92*	TraesCS6A02G390600	705	77,615.2	5.62	26.85	79.09	−0.201
*Ta4CL93*	TraesCS6B02G042400	556	60,106.14	5.37	40.23	91.35	0.088
*Ta4CL94*	TraesCS6B02G042900	555	59,711.82	5.76	36.82	92.92	0.125
*Ta4CL95*	TraesCS6B02G079800	592	62,860.4	7.94	38.07	87.4	0.057
*Ta4CL96*	TraesCS6B02G179900	337	36,543.7	5.84	30.85	104.18	0.116
*Ta4CL97*	TraesCS6B02G294100	573	60,767.1	5.46	33.47	99.42	0.224
*Ta4CL98*	TraesCS6B02G431000	572	63,611.64	6.01	24.87	81.43	−0.232
*Ta4CL99*	TraesCS6D02G141700	549	58,829.93	5.29	39.96	97.19	0.087
*Ta4CL100*	TraesCS6D02G248000	568	60,154.38	5.45	32.41	99.98	0.229
*Ta4CL101*	TraesCS6D02G376900	667	73,820	5.74	25.81	78.19	−0.215
*Ta4CL102*	TraesCS7A02G011700	1198	130,912.24	5.76	41.49	89.67	−0.022
*Ta4CL103*	TraesCS7A02G033700	1143	124,812.3	5.68	39.87	91.69	0.002
*Ta4CL104*	TraesCS7A02G310100	605	65,750.56	8.65	40.39	91.69	−0.023
*Ta4CL105*	TraesCS7A02G496200	557	59,429.4	5.31	36.56	97.38	0.116
*Ta4CL106*	TraesCS7B02G210000	591	63,758.29	8.25	42.28	90.9	0.069
*Ta4CL107*	TraesCS7B02G448600	1145	125,202.44	5.83	41.35	88.72	−0.05
*Ta4CL108*	TraesCS7D02G030200	891	97,629.47	5.68	39.26	91.14	−0.041
*Ta4CL109*	TraesCS7D02G306700	551	59,312.91	6.94	40.35	92.4	0.011
*Ta4CL110*	TraesCS7D02G483400	560	59,574.6	5.25	36.77	97.91	0.147

## Data Availability

Data are contained within the article and its Supplementary Materials.

## Supplementary Materials

The following supporting information can be downloaded at https://www.mdpi.com/article/10.3390/plants14091301/s1. Figure S1: Expression analysis of *Ta4CL91* in control and silenced wheat lines. Figure S2: Phenotypic comparison of control and *TaPDS*-silenced wheat plants. Table S1: Identified *4CL* gene family protein sequences in wheat. Table S2: Conserved motif sequences in wheat *4CL* family proteins. Table S3: Primers used in this study.

## References

[1] Shiferaw B., Smale M., Braun H.-J., Duveiller E., Reynolds M., Muricho G. (2013). Crops that feed the world 10. Past successes and future challenges to the role played by wheat in global food security. Food Secur..

[2] Dutta D., Karmakar S., Hossain A., Sadhukhan R., Atta K., Pramanick S., Khan M.K., Pandey A., Hamurcu M., Gupta O.P., Gezgin S. (2023). Chapter 1—Wheat and abiotic stress challenges: An overview. Abiotic Stresses in Wheat.

[3] Alam M.N., Islam M.Z., Farukh M.A., Chan Z., Akhter M.M., Abedin M.T., Hossain M.M. (2024). Detrimental effects of abiotic stress on wheat and its management techniques. Cereal Research Communications.

[4] Furtak K., Wolińska A. (2023). The impact of extreme weather events as a consequence of climate change on the soil moisture and on the quality of the soil environment and agriculture—A review. CATENA.

[5] Lavhale S.G., Kalunke R.M., Giri A.P. (2018). Structural, functional and evolutionary diversity of 4-coumarate-CoA ligase in plants. Planta.

[6] Hamberger B., Hahlbrock K. (2004). The *4-coumarate:CoA ligase* gene family in *Arabidopsis thaliana* comprises one rare, sinapate-activating and three commonly occurring isoenzymes. Proc. Natl. Acad. Sci. USA.

[7] Schneider K., Hövel K., Witzel K., Hamberger B., Schomburg D., Kombrink E., Stuible H.P. (2003). The substrate specificity-determining amino acid code of 4-coumarate:CoA ligase. Proc. Natl. Acad. Sci. USA.

[8] Stuible H., Büttner D., Ehlting J., Hahlbrock K., Kombrink E. (2000). Mutational analysis of *4-coumarate:CoA ligase* identifies functionally important amino acids and verifies its close relationship to other adenylate-forming enzymes. FEBS Lett..

[9] Stuible H.P., Kombrink E. (2001). Identification of the substrate specificity-conferring amino acid residues of 4-coumarate:coenzyme A ligase allows the rational design of mutant enzymes with new catalytic properties. J. Biol. Chem..

[10] De Azevedo Souza C., Barbazuk B., Ralph S.G., Bohlmann J., Hamberger B., Douglas C.J. (2008). Genome-wide analysis of a land plant-specific *acyl:coenzyme A synthetase (ACS)* gene family in *Arabidopsis*, poplar, rice and Physcomitrella. New Phytol..

[11] Ehlting J., Büttner D., Wang Q., Douglas C.J., Somssich I.E., Kombrink E. (1999). Three 4-coumarate:coenzyme A ligases in *Arabidopsis thaliana* represent two evolutionarily divergent classes in angiosperms. Plant J..

[12] Li Y., Kim J.I., Pysh L., Chapple C. (2015). Four Isoforms of Arabidopsis 4-Coumarate:CoA Ligase Have Overlapping yet Distinct Roles in Phenylpropanoid Metabolism. Plant Physiol..

[13] Sun H., Li Y., Feng S., Zou W., Guo K., Fan C., Si S., Peng L. (2013). Analysis of five rice 4-coumarate:coenzyme A ligase enzyme activity and stress response for potential roles in lignin and flavonoid biosynthesis in rice. Biochem. Biophys. Res. Commun..

[14] Chowdhury M.E.K., Choi B., Cho B.-K., Kim J.B., Park S.U., Natarajan S., Lim H.-S., Bae H. (2013). Regulation of 4CL, encoding 4-coumarate: Coenzyme A ligase, expression in kenaf under diverse stress conditions. Plant Omics.

[15] Moura J.C., Bonine C.A., de Oliveira Fernandes Viana J., Dornelas M.C., Mazzafera P. (2010). Abiotic and biotic stresses and changes in the lignin content and composition in plants. J. Integr. Plant Biol..

[16] Cesarino I. (2019). Structural features and regulation of lignin deposited upon biotic and abiotic stresses. Curr. Opin. Biotechnol..

[17] Wang M., Zhang Y., Zhu C., Yao X., Zheng Z., Tian Z., Cai X. (2021). EkFLS overexpression promotes flavonoid accumulation and abiotic stress tolerance in plant. Physiol. Plant..

[18] Shomali A., Das S., Arif N., Sarraf M., Zahra N., Yadav V., Aliniaeifard S., Chauhan D.K., Hasanuzzaman M. (2022). Diverse Physiological Roles of Flavonoids in Plant Environmental Stress Responses and Tolerance. Plants.

[19] Li X.-l., LÜ X., Wang X.-h., Peng Q., Zhang M.-s., Ren M.-j. (2020). Biotic and abiotic stress-responsive genes are stimulated to resist drought stress in purple wheat. J. Integr. Agric..

[20] Gulzar F., Yang H., Chen J., Hassan B., Huang X., Qiong F. (2024). 6-BA Reduced Yield Loss under Waterlogging Stress by Regulating the Phenylpropanoid Pathway in Wheat. Plants.

[21] Chen X., Su W., Zhang H., Zhan Y., Zeng F. (2020). Fraxinus mandshurica 4-coumarate-CoA ligase 2 enhances drought and osmotic stress tolerance of tobacco by increasing coniferyl alcohol content. Plant Physiol. Biochem. PPB.

[22] Chen X., Wang H., Li X., Ma K., Zhan Y., Zeng F. (2019). Molecular cloning and functional analysis of 4-Coumarate:CoA ligase 4(4CL-like 1)from *Fraxinus mandshurica* and its role in abiotic stress tolerance and cell wall synthesis. BMC Plant Biol..

[23] Wang C.H., Yu J., Cai Y.X., Zhu P.P., Liu C.Y., Zhao A.C., Lü R.H., Li M.J., Xu F.X., Yu M.D. (2016). Characterization and Functional Analysis of 4-Coumarate:CoA Ligase Genes in Mul-berry. PLoS ONE.

[24] Sun S.C., Xiong X.P., Zhang X.L., Feng H.J., Zhu Q.H., Sun J., Li Y.J. (2020). Characterization of the Gh4CL gene family reveals a role of Gh4CL7 in drought tolerance. BMC Plant Biol..

[25] Lee D., Ellard M., Wanner L.A., Davis K.R., Douglas C.J. (1995). The *Arabidopsis thaliana* 4-coumarate:CoA ligase (4CL) gene: Stress and developmentally regulated expression and nucleotide sequence of its cDNA. Plant Mol. Biol..

[26] Douglas C.J. (1996). Phenylpropanoid metabolism and lignin biosynthesis: From weeds to trees. Trends Plant Sci..

[27] Yang M., Chen S., Chao K., Ji C., Shi Y. (2024). Effects of nano silicon fertilizer on the lodging resistance characteristics of wheat basal second stem node. BMC Plant Biol..

[28] Hu Y., Qin F., Wu Z., Wang X., Ren X., Jia Z., Wang Z., Chen X., Cai T. (2024). Heterogeneous population distribution enhances resistance to wheat lodging by optimizing the light environment. J. Integr. Agric..

[29] Cao Y., Han Y., Li D., Lin Y., Cai Y. (2016). Systematic Analysis of the 4-Coumarate:Coenzyme A Ligase (4CL) Related Genes and Expression Profiling during Fruit Development in the Chinese Pear. Genes.

[30] Shockey J.M., Fulda M.S., Browse J. (2003). Arabidopsis contains a large superfamily of acyl-activating enzymes. Phylogenetic and biochemical analysis reveals a new class of acyl-coenzyme a synthetases. Plant Physiol..

[31] ul Haq S., Khan A., Ali M., Khattak A.M., Gai W.-X., Zhang H.-X., Wei A.-M., Gong Z.-H. (2019). Heat Shock Proteins: Dynamic Biomolecules to Counter Plant Biotic and Abiotic Stresses. Int. J. Mol. Sci..

[32] Hu C., Yang J., Qi Z., Wu H., Wang B., Zou F., Mei H., Liu J., Wang W., Liu Q. (2022). Heat shock proteins: Biological functions, pathological roles, and therapeutic opportunities. MedComm.

[33] Hong-Bo S., Zong-Suo L., Ming-An S. (2005). LEA proteins in higher plants: Structure, function, gene expression and regulation. Colloids Surf. B Biointerfaces.

[34] Salgotra R.K., Chauhan B.S. (2023). Genetic Diversity, Conservation, and Utilization of Plant Genetic Resources. Genes.

[35] Abhinandan K., Skori L., Stanic M., Hickerson N.M.N., Jamshed M., Samuel M.A. (2018). Abiotic Stress Signaling in Wheat—An Inclusive Overview of Hormonal Interactions During Abiotic Stress Responses in Wheat. Front. Plant Sci..

[36] Hossain A., Skalicky M., Brestic M., Maitra S., Ashraful Alam M., Syed M.A., Hossain J., Sarkar S., Saha S., Bhadra P. (2021). Consequences and Mitigation Strategies of Abiotic Stresses in Wheat (*Triticum aestivum* L.) under the Changing Climate. Agronomy.

[37] Zhang C.H., Ma T., Luo W.C., Xu J.M., Liu J.Q., Wan D.S. (2015). Identification of 4CL Genes in Desert Poplars and Their Changes in Expression in Response to Salt Stress. Genes.

[38] Hernandez-Garcia C.M., Finer J.J. (2014). Identification and validation of promoters and cis-acting regulatory elements. Plant Sci..

[39] Danquah A., de Zelicourt A., Colcombet J., Hirt H. (2014). The role of ABA and MAPK signaling pathways in plant abiotic stress responses. Biotechnol. Adv..

[40] Zhang J., Jia W., Yang J., Ismail A.M. (2006). Role of ABA in integrating plant responses to drought and salt stresses. Field Crops Res..

[41] Zhang J., Yu D., Zhang Y., Liu K., Xu K., Zhang F., Wang J., Tan G., Nie X., Ji Q. (2017). Vacuum and Co-cultivation Agroinfiltration of (Germinated) Seeds Results in Tobacco Rattle Virus (TRV) Mediated Whole-Plant Virus-Induced Gene Silencing (VIGS) in Wheat and Maize. Front. Plant Sci..

[42] Wang Q., Yu F., Xie Q. (2020). Balancing growth and adaptation to stress: Crosstalk between brassinosteroid and abscisic acid signaling. Plant Cell Environ..

[43] Kudo M., Kidokoro S., Yoshida T., Mizoi J., Todaka D., Fernie A.R., Shinozaki K., Yamaguchi-Shinozaki K. (2017). Double overexpression of DREB and PIF transcription factors improves drought stress tolerance and cell elongation in transgenic plants. Plant Biotechnol. J..

[44] Mistry J., Chuguransky S., Williams L., Qureshi M., Salazar G.A., Sonnhammer E.L.L., Tosatto S.C.E., Paladin L., Raj S., Richardson L.J. (2021). Pfam: The protein families database in 2021. Nucleic Acids Res..

[45] Kumar S., Stecher G., Tamura K. (2016). MEGA7: Molecular Evolutionary Genetics Analysis Version 7.0 for Bigger Datasets. Mol. Biol. Evol..

[46] Wilkins M.R., Gasteiger E., Bairoch A., Sanchez J.C., Williams K.L., Appel R.D., Hochstrasser D.F. (1999). Protein identification and analysis tools in the ExPASy server. Methods Mol. Biol..

[47] Chen C., Chen H., Zhang Y., Thomas H.R., Frank M.H., He Y., Xia R. (2020). TBtools: An Integrative Toolkit Developed for Interactive Analyses of Big Biological Data. Mol. Plant.

[48] Bailey T.L., Boden M., Buske F.A., Frith M., Grant C.E., Clementi L., Ren J., Li W.W., Noble W.S. (2009). MEME SUITE: Tools for motif discovery and searching. Nucleic Acids Res..

[49] Marchler-Bauer A., Bryant S.H. (2004). CD-Search: Protein domain annotations on the fly. Nucleic Acids Res..

[50] Rombauts S., Déhais P., Van Montagu M., Rouzé P. (1999). PlantCARE, a plant cis-acting regulatory element database. Nucleic Acids Res..

[51] (2014). A chromosome-based draft sequence of the hexaploid bread wheat (*Triticum aestivum*) genome. Science.

[52] Pfaffl M.W. (2001). A new mathematical model for relative quantification in real-time RT-PCR. Nucleic Acids Res..

[53] Sade N., Vinocur B.J., Diber A., Shatil A., Ronen G., Nissan H., Wallach R., Karchi H., Moshelion M. (2009). Improving plant stress tolerance and yield production: Is the tonoplast aquaporin SlTIP2;2 a key to isohydric to anisohydric conversion?. New Phytol..

