# Supplementary material for: Characterization of 4-Coumarate-CoA Ligase (4CL) Genes in Wheat Uncovers Ta4CL91’s Role in Drought and Salt Stress Adaptation
Source: Plants (Basel). 2025 Apr 25;14(9):1301. doi: 10.3390/plants14091301 (PMC12073920; doi:10.3390/plants14091301)
Supplement: Supplementary file 1 [file plants-14-01301-s001.zip › Fig. S1.pdf]

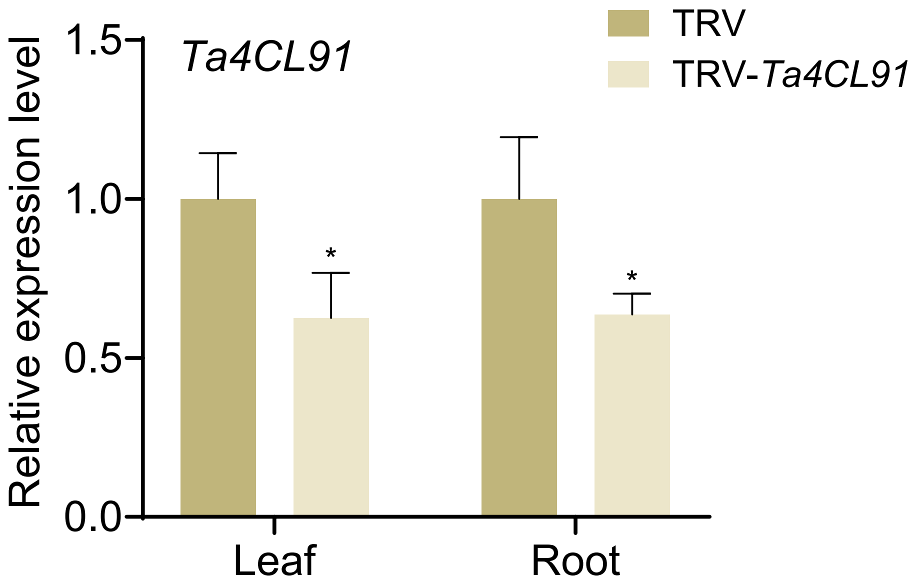

Figure S1. Expression analysis of *Ta4CL91* in control and silenced wheat lines. To investigate the expression levels of *Ta4CL91* in both control and *Ta4CL91*-silenced lines, RT-qPCR analysis was performed. Leaf and root tissues were harvested from the control and silenced lines, and the relative expression of *Ta4CL91* was quantified. Statistical significance was assessed using Student's *t*-test, with significance levels set at $*P < 0.05$ and $**P < 0.01$.
