# Supplementary material for: Characterization of 4-Coumarate-CoA Ligase (4CL) Genes in Wheat Uncovers Ta4CL91’s Role in Drought and Salt Stress Adaptation
Source: Plants (Basel). 2025 Apr 25;14(9):1301. doi: 10.3390/plants14091301 (PMC12073920; doi:10.3390/plants14091301)
Supplement: Supplementary file 1 [file plants-14-01301-s001.zip › Fig. S2.pdf]

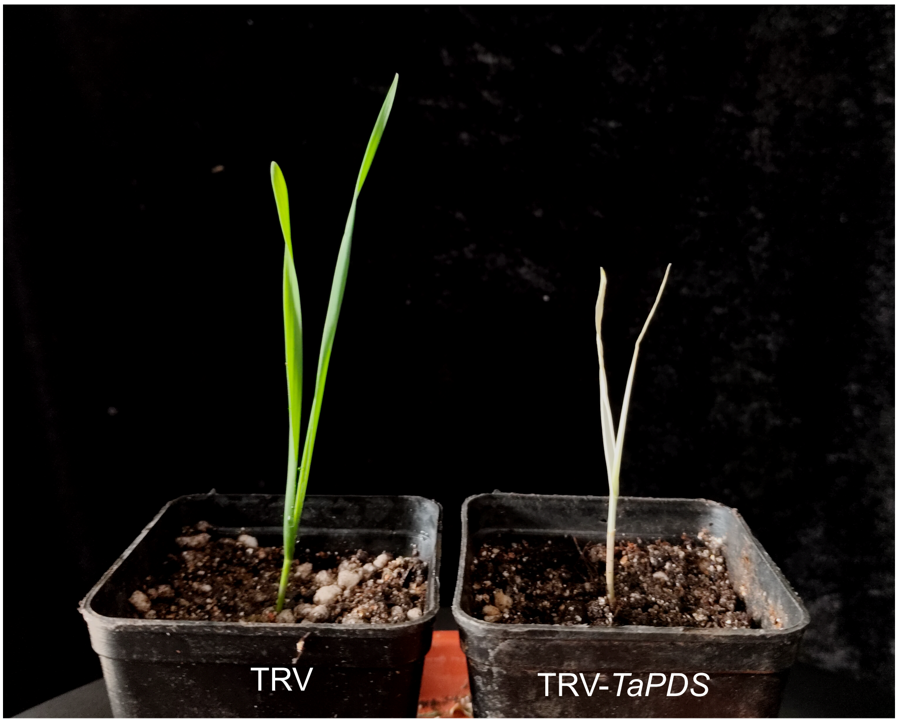

Figure S2. Phenotypic comparison of control and *TaPDS*-silenced wheat plants. Seedlings infected with TRV-*TaPDS* exhibited a distinct albino phenotype.
